# Long-term experience with octreotide and lanreotide for the treatment of gastroenteropancreatic neuroendocrine tumors

**DOI:** 10.1007/s12094-024-03732-w

**Published:** 2024-09-24

**Authors:** Barbara Kiesewetter, Friedrich Franz Pflüger, Philipp Melhorn, Peter Mazal, Markus Raderer

**Affiliations:** 1 Department of Medicine I, Division of Oncology, Medical University of Vienna, Waehringer Guertel 18–20, A-1090 Vienna, Austria; 2 Department of Pathology, Medical University of Vienna, Vienna, Austria

**Keywords:** Neuroendocrine neoplasms, Somatostatin receptor, Somatostatin analogs, Prognosis, Real-world study

## Abstract

**Introduction:**

The somatostatin analogs (SSA) octreotide and lanreotide are a mainstay in the treatment of neuroendocrine tumors (NET). The two pivotal trials differed considerably in terms of patient characteristics and are not directly comparable. Further comparative data are lacking.

**Methods:**

This retrospective chart review study included patients with gastroenteropancreatic NET grade 1 or 2 who were treated with octreotide LAR or lanreotide autogel. The main aim was to compare the two SSA based on progression-free survival (PFS) and overall survival (OS) from treatment start.

**Results:**

In total, 129 patients were analyzed, 60% (n = 77) had a small intestinal NET and 31% (n = 40) a pancreatic NET. Histologically, 34% (n = 44) had NET G1, 55% (n = 71) a NET G2, and 11% (n = 14) a NET G1/G2 unclassified. Lanreotide was used in 90 patients (70%) and octreotide in 39 patients (30%). Overall, the median PFS was 32.2 months (95% CI 23.0–42.9 months). No PFS difference (p = 0.8) was observed between lanreotide (29.8 months, 95% CI 18.7–48.5 months) and octreotide (36.0 months, 95% CI 23.2–68.2 months). Median OS from treatment start was calculated at 93.5 months (95% CI 71.1–132.9 months). Again, the median OS following lanreotide (113.4 months, 95% CI 62.3–NA months) or after octreotide (90.3 months, 95% CI 71.1–NA months) did not differ significantly (p > 0.9).

**Conclusions:**

Our long-term experience with octreotide and lanreotide in NET did not reveal differences in antitumor effectiveness. This is consistent with previous reports and might suggest that both SSA can be used interchangeably if needed.

## Introduction

Neuroendocrine tumors (NET) are malignant tumors arising from endocrine cells and are commonly found in the lung, pancreas, and gastrointestinal tract [1]. NET of gastroenteropancreatic origin (GEP-NET) typically show a rather indolent disease course with a favorable prognosis [2]. This is in line with the comparatively low proliferative activity (measured by the Ki-67 index) seen in NET grade 1 (G1, Ki-67 < 3%) and NET G2 (Ki-67 3–20%) [3]. In contrast, NET G3 and neuroendocrine carcinoma (NEC) are highly proliferative (with a Ki-67 index > 20% or > 10 mitoses/2 mm^2^) and often rapidly progressing [3, 4].

Somatostatin analogs (SSA) are a standard therapy for the treatment of metastatic NET G1/G2 [5, 6]. SSA target the somatostatin receptors (SSTR) that are present in most NET but also found in other tumors and physiologically expressed in several organs [7, 8]. Like the peptide hormone somatostatin, the synthetic SSA exert pleiotropic inhibitory effects on various hormones and gastrointestinal processes [9]. Consequently, it has long been used for symptom control in NET patients that exhibit hormonal syndromes like carcinoid syndrome (marked by flushing, diarrhea, and/or bronchospasm) [10].

Apart from symptom control, somatostatin analogs are approved drugs for tumor control in NET [11]. The phase III trials PROMID [12] (octreotide) and CLARINET [13] (lanreotide) demonstrated the efficacy of SSA in delaying disease progression compared to placebo (median time to tumor progression [TTP] of 14.3 versus 6 months and median progression-free survival [PFS] not reached versus 18.0 months, respectively). To date, no overall survival (OS) benefit has been established – likely a result of the cross-over design of these studies [13, 14]. Furthermore, substantial differences between the two study populations make comparisons difficult [12, 13]. Head-to-head clinical trials are lacking, and few studies provided comparative outcome data for these two compounds [15, 16]. Their antiproliferative efficacy is generally considered to be equal (“drug class effect”) [17], and they have been used interchangeably despite small differences in approval [15]. From a pharmacological perspective, octreotide and lanreotide have a similar receptor binding affinity but different pharmacokinetic profiles and routes of administration [7, 18]. SSA are usually well tolerated (with gastrointestinal adverse effects being the most frequent) [11], and their safety profile compares favorably to other treatments endorsed for NET, including peptide receptor radionuclide therapy (PPRT), everolimus, sunitinib, and chemotherapy [5].

Given the lack of comparative studies of octreotide and lanreotide, our aim was to compare survival outcomes of these two therapies in GEP-NET patients treated at our institution, a European Neuroendocrine Tumor Society (ENETS) certified Center of Excellence. Addressing this gap might improve the management of NET by providing clearer guidance on therapeutic decision-making.

## Methods

This single-center analysis was designed as a retrospective chart review study, including NET patients treated at the Medical University of Vienna between January 2010 and June 2020. The inclusion criteria were histologically verified NET G1 or NET G2 of (presumed) gastroenteropancreatic origin, therapy with long-acting SSA (octreotide LAR or lanreotide autogel), and age ≥ 18. The exclusion criteria were NET G3 or NEC, pulmonary origin, and insufficient available patient data. Furthermore, short-acting SSA formulations for functional control were omitted from this analysis. Patient data were extracted from electronic health records (EHR) and populated into a secure database built with FileMaker (Claris International Inc., Santa Clara, CA, USA). The relevant data included basic patient information (age, sex, symptoms, and Eastern Cooperative Oncology Group [ECOG] status), NET-related parameters (primary localization, disease stage, functional status, and chromogranin A levels), treatment information (SSA dose and interval and adverse events), and outcome data (PFS, OS, and death). This study was approved by the Ethics Committee of the Medical University of Vienna (EK No: 1743/2020). Due to the retrospective approach of the study, it was not necessary to obtain specific informed consent according to local guidelines.

The primary objective was to calculate the PFS following SSA therapy. The secondary endpoint was to assess OS from treatment start and diagnosis. Exploratory endpoints included descriptive assessment of the patient cohort and of adverse events as well as an evaluation of potentially prognostic factors. PFS was defined as the duration between treatment initiation and disease progression or death, and patients were censored at the date of last patient contact if no progression event was recorded. In comparison, OS was the period from the treatment start date (or date of diagnosis) to the date of death. The last follow-up occurred March 13, 2023. Disease progression was determined based on routine radiological assessment performed depending on the clinical case at intervals of about 3–6 months as per guideline recommendations. Additional check-ups with clinical assessment and biochemical follow-up examinations were carried out according to individual requirements, e.g., in the event of uncontrolled hormonal symptoms.

For the statistical analysis, the programming language R version 4.3.2 (R Foundation for Statistical Computing, Vienna, Austria) was used with the packages tidyverse [19], ggsurvfit [20], gtsummary [21], gt [22], and survival [23]. Descriptive statistics are given as medians and ranges (for quantitative variables) and as percentages and frequencies (for categorical variables). To calculate PFS and OS curves, the Kaplan–Meier method was employed. Subgroups were compared using the log-rank test. Univariate and multivariable Cox regression analysis was used to assess the prognostic value of relevant factors. In a multivariable Cox model, the effect of SSA type was adjusted for clinically important or statistically significant factors from the univariate analysis (keeping an events-per-variable ratio > 15). To compare subgroups, Fisher’s exact test, Wilcoxon rank sum test, and Kruskal–Wallis rank sum test were used. The significance level was set to 0.05. The reverse Kaplan–Meier estimator was used to determine median follow-up [24].

## Results

### Patient characteristics

The full analysis set comprised 129 patients treated with SSA for gastroenteropancreatic NET. Sex was roughly balanced with 71 male (55%) and 58 female (45%) patients. The median age at diagnosis was 62 years (range: 27–84 years). Baseline ECOG status was unimpaired in 94% of patients (n = 109/116), and seven (6.0%) had ECOG 1. The majority of patients had a primary tumor in the small intestine (siNET, n = 77, 60%), followed by pancreatic NET (panNET, n = 40, 31%), and other locations (n = 12, 9.3%), i.e., tumors originating from the stomach (n = 1), rectum (n = 1), colon (n = 2), and from an unknown primary (CUP, n = 8). Overall, 44 patients (34%) had NET G1, 71 (55%) NET G2, and 14 (11%) a well-differentiated NET G1/G2 unclassified. The proportion of NET G1 was higher in siNET (43%) than in panNET (20%), and there was a significant difference in grade overall between the localizations (p = 0.025). The median Ki-67 index was 4%, with significant differences (p = 0.002) between siNET (3%), panNET (6%), and other sites (4%). A high fraction (23%) of panNET patients had a Ki-67 index > 10% (versus 4.3% of siNET patients). Stage IV disease was present at diagnosis in 98 patients (83%), with differences in disease stage between primary tumor sites (p = 0.006, stage IV in 90% of siNET versus 68% of panNET). All but three patients (98%) had distant metastatic disease at any time point, and the three had local lymph node metastases. Hormone secretion was present in 54% of patients (n = 61), with a difference (p < 0.001) between siNET (n = 52, 70%) and panNET (n = 4, 15%). On SSTR imaging, 100/101 (99%) were positive, the negative patient was immunohistochemically positive. Primary tumor resection was performed in 94/129 patients (73%) and was more frequent in siNET (86% versus 58% in panNET, p < 0.001), see Table 1.

**Table 1:** General patient characteristics

	Overall		By localization				By treatment type		
Variable	N	N = 129^*1*^	Small intestine, N = 77^*1*^	Pancreas, N = 40^*1*^	Other, N = 12^*1*^	p-value^*2*^	Lanreotide, N = 90^*1*^	Octreotide, N = 39^*1*^	p-value^*3*^
Sex	129					0.5			0.4
Female		58 (45%)	32 (42%)	19 (48%)	7 (58%)		38 (42%)	20 (51%)	
Male		71 (55%)	45 (58%)	21 (53%)	5 (42%)		52 (58%)	19 (49%)	
Age at diagnosis	129	62 (27, 84)	64 (27, 84)	62 (29, 82)	63 (54, 78)	0.7	63 (27, 82)	62 (29, 84)	0.7
ECOG performance score	116					0.2			0.2
0		109 (94%)	69 (95%)	32 (97%)	8 (80%)		78 (96%)	31 (89%)	
1		7 (6.0%)	4 (5.5%)	1 (3.0%)	2 (20%)		3 (3.7%)	4 (11%)	
Grading	129					0.025			> 0.9
NET G1		44 (34%)	33 (43%)	8 (20%)	3 (25%)		30 (33%)	14 (36%)	
NET G2		71 (55%)	40 (52%)	24 (60%)	7 (58%)		50 (56%)	21 (54%)	
NET unclassified (G1/G2)		14 (11%)	4 (5.2%)	8 (20%)	2 (17%)		10 (11%)	4 (10%)	
Ki-67 index (%)	110	4.0 (0.5, 19.0)	3.0 (0.5, 15.0)	6.0 (1.0, 19.0)	4.0 (1.0, 19.0)	0.002	5.0 (1.0, 19.0)	3.3 (0.5, 15.0)	0.067
Ki-67 > 10%	110	11 (10%)	3 (4.3%)	7 (23%)	1 (10%)	0.016	10 (13%)	1 (2.9%)	0.2
Disease stage	118					0.006			0.8
I		3 (2.5%)	0 (0%)	3 (8.1%)	0 (0%)		2 (2.5%)	1 (2.7%)	
II		8 (6.8%)	1 (1.4%)	6 (16%)	1 (10%)		7 (8.6%)	1 (2.7%)	
III		9 (7.6%)	6 (8.5%)	3 (8.1%)	0 (0%)		6 (7.4%)	3 (8.1%)	
IV		98 (83%)	64 (90%)	25 (68%)	9 (90%)		66 (81%)	32 (86%)	
Metastasized at any time point	129	126 (98%)	76 (99%)	38 (95%)	12 (100%)	0.5	88 (98%)	38 (97%)	> 0.9
Functionality	112	61 (54%)	52 (70%)	4 (15%)	5 (45%)	< 0.001	37 (48%)	24 (69%)	0.065
SSTR positive (before treatment)	101	100 (99%)	65 (98%)	24 (100%)	11 (100%)	> 0.9	70 (99%)	30 (100%)	> 0.9
Primary tumor resection	129	94 (73%)	66 (86%)	23 (58%)	5 (42%)	< 0.001	60 (67%)	34 (87%)	0.018
SSA as first systemic therapy	129					0.5			0.5
SSA first-line		107 (83%)	65 (84%)	31 (78%)	11 (92%)		73 (81%)	34 (87%)	
SSA later-line		22 (17%)	12 (16%)	9 (23%)	1 (8.3%)		17 (19%)	5 (13%)	
SSA type administered	129					0.027			
Lanreotide		90 (70%)	47 (61%)	32 (80%)	11 (92%)				
Octreotide		39 (30%)	30 (39%)	8 (20%)	1 (8.3%)				
Treatment setting	129								
Palliative		129 (100%)	77 (100%)	40 (100%)	12 (100%)		90 (100%)	39 (100%)	
Disease setting	129					0.084			> 0.9
Locally advanced		7 (5.4%)	2 (2.6%)	5 (13%)	0 (0%)		5 (5.6%)	2 (5.1%)	
Metastasized		122 (95%)	75 (97%)	35 (88%)	12 (100%)		85 (94%)	37 (95%)	
ECOG at treatment start	106					0.5			> 0.9
0		94 (89%)	67 (91%)	17 (85%)	10 (83%)		64 (89%)	30 (88%)	
1		12 (11%)	7 (9.5%)	3 (15%)	2 (17%)		8 (11%)	4 (12%)	
Chromogranin A elevated	79	57 (72%)	37 (74%)	14 (67%)	6 (75%)	0.9	40 (73%)	17 (71%)	> 0.9

^1^n (%); Median (Range)

^2^Fisher’s exact test; Kruskal–Wallis rank sum test

^3^Fisher’s exact test; Wilcoxon rank sum test

### Treatment information

**Fig. 1:**
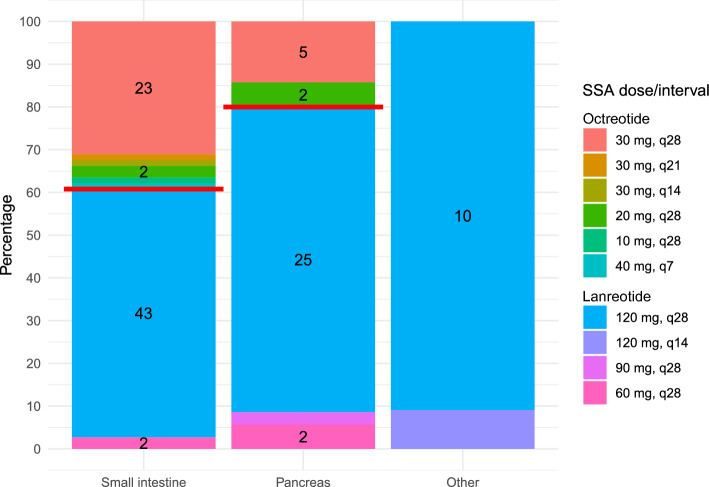
Percentages (y-axis) and frequencies (numbers in bars) of somatostatin analog doses and intervals shown for different primary tumor localization

Two thirds of patients received treatment with lanreotide (n = 90, 70%) and one third with octreotide (n = 39, 30%). While octreotide was commonly used in siNET (39% versus 61% lanreotide), lanreotide was the preferred SSA for panNET (20% versus 80% lanreotide), with these differences reaching statistical significance (p = 0.027). There was no difference in the type of SSA administered between NET G1 (68% lanreotide), NET G2 (70%), and NET unclassified (71%) with p > 0.9. SSA was the first systemic treatment in 107/129 patients (83%), with previous treatments including PRRT (n = 11), everolimus (n = 5), capecitabine/temozolomide (n = 2), cisplatin/etoposide (n = 1), PRRT + capecitabine (n = 1), FOLFIRINOX (n = 1), clinical trial drugs (n = 3), interferon + SSA (n = 1), and interferon (n = 1). The applied SSA dose was documented in 120 cases, see Fig. 1. Among the 39 patients treated with octreotide, 28/39 (71.8%) received 30 mg every 28 days, 4/39 (10.3%) 20 mg every 28 days, and one patient each (2.6%) 10 mg every 28, 30 mg every 21, 30 mg every 14, and 40 mg every 7 days. Out of the 90 patients treated with lanreotide, 78/90 (86.7%) received 120 mg every 28 days, 4/90 (4.4%) 60 mg every 28 days, and one patient each (1.1%) received 90 mg every 28 and 120 mg every 14 days. In total, 82.2% of patients (106/129) received SSA therapy at the approved dose for disease control, whereas 14/129 (10.9%) were treated with a lower dose or with a higher dose / shorter interval for better symptom control. During treatment, there was a dose increase in 6 cases and an interval reduction for better symptom control in 5 cases. At SSA initiation, 95% (n = 122) were metastasized, and 5.4% (n = 7) had locally advanced disease. All patients received palliative SSA therapy. At SSA start, 94/106 patients (89%) had an ECOG 0 and 12/106 (11%) had ECOG 1. Pretreatment chromogranin A was elevated in 57/79 patients (72%). There were only minor differences in baseline characteristics between patients treated with octreotide versus lanreotide, including a lower median Ki-67 index in patients receiving octreotide (3.3% versus 5%), a higher percentage with hormonal symptoms (69% versus 48%), and a higher fraction with tumor resection (87% versus 67%), see Table 1.

### Survival outcome

**Fig. 2:**
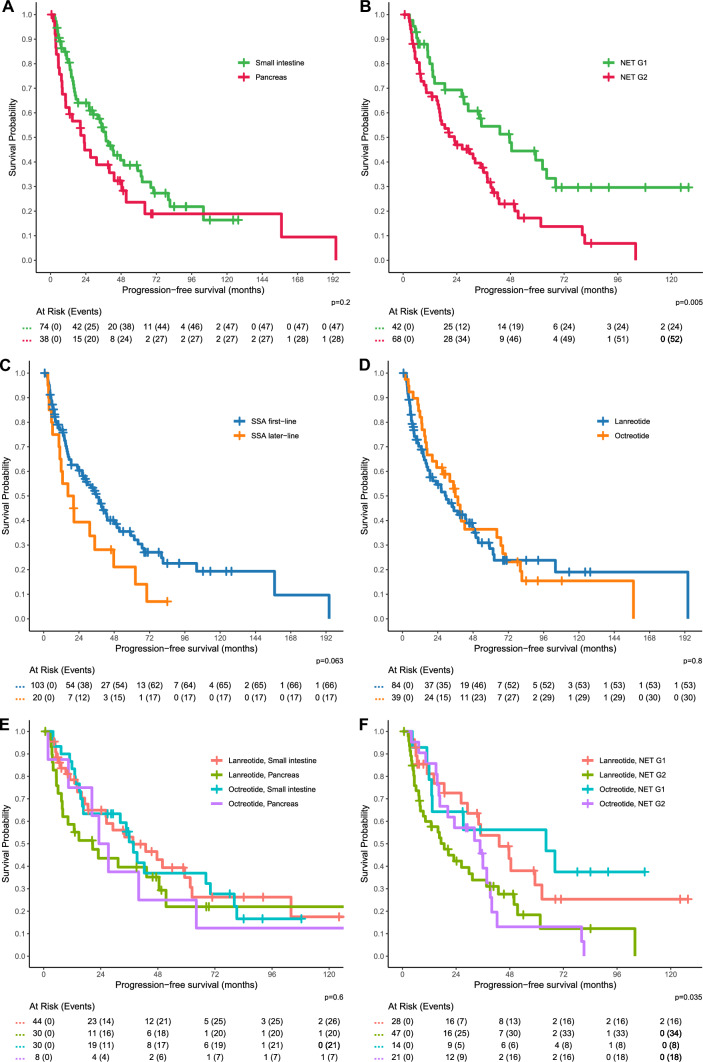
Kaplan–Meier analysis of progression-free survival with comparisons between primary localizations (**A**), tumor grades (**B**), first-line and later-line SSA (**C**), between lanreotide and octreotide (**D**), based on SSA type and localization (**E**), and based on SSA type and tumor grade (**F**)

In the progression-free survival analysis, 123 patients could be included, and 84 events (progression or death) were recorded. The median PFS was 32.2 months (95% CI 23.0–42.9 months). Numerically different (p = 0.3) median PFS durations were observed for the localizations small intestinal NET (37.7 months, 95% CI 29.1–61.6 months), pancreatic NET (23.0 months, 95% CI 10.4–48.5 months), and other sites (18.7 months, 95% CI 16.5–NA months), see Fig. 2A for a comparison of siNET and panNET (p = 0.2). There was a significant difference (p = 0.005) between NET G1 and NET G2 with a median PFS of 47.6 months (95% CI 29.1–NA months) and 23.0 months (95% CI 16.3–37.6 months), respectively, see Fig. 2B. Receiving SSA as a first-line treatment resulted in a longer PFS compared to SSA as a later-line therapy (p = 0.063, median PFS 36.0 months with 95% CI 26.4–49.9 months versus 18.4 months with 95% CI 11.0–62.4 months), see Fig. 2C. With respect to SSA type, the median PFS achieved with lanreotide was 29.8 months (95% CI 18.7–48.5 months) and with octreotide 36.0 months (95% CI 23.2–68.2 months). The log-rank test yielded a p-value of 0.8, indicating that neither of the two compounds was superior regarding PFS, see Fig. 2D. The rates of progression-free survival at 6 months were 83.1% and 92.3%, respectively, at 12 months 70.2% and 82.1%, respectively, and at 24 months 54.7% and 61.5%, respectively. Stratified by primary tumor site, the median PFS for lanreotide in panNET was 20.6 months (95% CI 8.0–NA months) and for octreotide 25.2 months (95% CI 20.3–NA months), with a p-value of 0.8. In siNET, the median PFS for lanreotide (37.6 months, 95% CI 26.2–62.4 months) was similar (p > 0.9) to that for octreotide (37.7 months, 95% CI 16.7–80.1 months), see Fig. 2E. With respect to grading, the median PFS with lanreotide in NET G1 was 43.3 months (95% CI 29.1–NA months) and with octreotide 64.2 months (95% CI 13.3–NA months). In comparison, lanreotide achieved a median PFS of 18.7 months in NET G2 (95% CI 9.3–37.6 months) and octreotide a median PFS of 34.7 months (95% CI 16.7–42.3 months), see Fig. 2F. While there was a PFS difference based on grade and SSA overall (p = 0.035), there was none for NET G1 (p = 0.6) and NET G2 (p = 0.8) separately.

The median PFS of patients with a high Ki-67 index (> 10%) was almost a third of the median PFS of patients with a lower Ki-67 index, i.e., 12.8 months (95% CI 4.8–NA months) versus 34.1 months (95% CI 23.2–43.3 months), with p = 0.072. There was no difference in PFS (p = 0.6) between functional and non-functional NET, with a median PFS of 36.0 months (95% CI 18.7–59.3 months) and 34.1 months (95% CI 20.6–51.5 months), respectively. Furthermore, no difference was observed between NET with normal versus elevated pretreatment chromogranin A levels (p = 0.7), the median PFS being 37.7 months (95% CI 20.6–NA months) and 35.0 months (95% CI 18.7–51.5 months), respectively. In Cox regression, NET G2 was associated with a significantly shorter PFS with a hazard ratio (HR) of 1.98 (95% CI 1.21–3.24) in univariate analysis and 1.78 (95% CI 1.08–2.95) in multivariable analysis, indicating up to twice the risk of progression or death, see Table 2. Furthermore, a history of primary tumor resection was a favorable prognostic factor (HR 0.58, 95% CI 0.36–0.92), albeit only in univariate analysis. Sex, primary tumor site, functional symptoms, SSA type, ECOG at treatment start, and chromogranin A elevation were not prognostic for PFS, but there was a trend for high Ki-67 (> 10%) and SSA treatment line (first versus later line) to be prognostically relevant (p = 0.077 and p = 0.065, respectively), see Table 2.

**Table 2:** Cox regression model for progression-free survival

	Univariate				Multivariable		
Characteristic	N	HR^*1*^	95% CI^*1*^	p-value	HR^*1*^	95% CI^*1*^	p-value
Sex	123						
* Female*		–	–				
* Male*		1.19	0.77, 1.85	0.4			
Histology	123						
* NET G1*		–	–		–	–	
* NET G2*		1.98	1.21, 3.24	0.006	1.78	1.08, 2.95	0.025
* NET unclassified (G1/G2)*		0.60	0.24, 1.47	0.3	0.43	0.17, 1.12	0.085
Ki-67 index > 10%	105						
* No*		–	–				
* Yes*		1.95	0.93, 4.11	0.077			
Primary tumor site	123						
* Other*		–	–		–	–	
* Pancreas*		0.94	0.43, 2.07	0.9	0.76	0.33, 1.71	0.5
* Small intestine*		0.67	0.32, 1.42	0.3	0.49	0.21, 1.14	0.10
Surgery	123	0.58	0.36, 0.92	0.020	0.76	0.45, 1.29	0.3
Functional symptoms	108						
* No*		–	–				
* Yes*		0.89	0.55, 1.44	0.6			
SSA as first line	123						
* SSA first-line*		–	–		–	–	
* SSA later-line*		1.66	0.97, 2.83	0.065	1.61	0.93, 2.80	0.087
SSA type	123						
* Lanreotide*		–	–		–	–	
* Octreotide*		0.93	0.59, 1.46	0.8	1.14	0.70, 1.86	0.6
ECOG at treatment start	103						
* 0*		–	–				
* 1*		0.91	0.39, 2.12	0.8			
Chromogranin A elevated	76						
* No*		–	–				
* Yes*		1.16	0.58, 2.34	0.7			

^*1*^*HR* hazard ratio, *CI* confidence interval

**Fig. 3:**
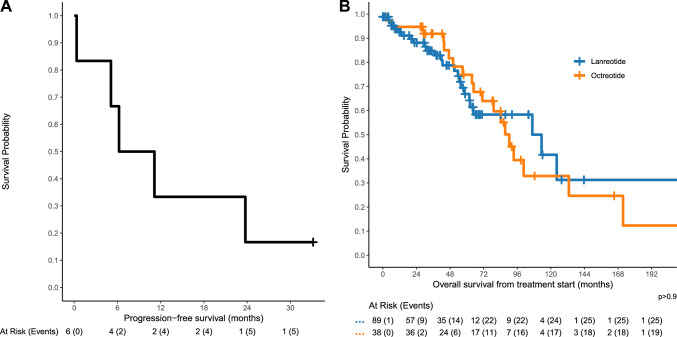
PFS after restart of SSA (**A**) and OS from first SSA treatment start (**B**)

Six patients had a second line of treatment with SSA (i.e., following disease progression; excluding maintenance therapy after PRRT and switching of SSA formulations), with further progression observed in five cases. Of those patients, two received lanreotide and four octreotide. The median PFS was 8.7 months (95% CI 5.1–NA months), see Fig. 3A.

According to reverse Kaplan–Meier, the median follow-up from SSA start was 64.0 months (over 5.3 years), with a significant difference (p < 0.001) between octreotide (92.0 months) and lanreotide (47.3 months). The median follow-up from diagnosis was 91.2 months (7.6 years). The median OS from treatment start was 93.5 months (95% CI 71.1–132.9 months), with no significant difference (p > 0.9) between lanreotide (median OS 113.4 months, 95% CI 62.3–NA months) and octreotide (median OS 90.3 months, 95% CI 71.1–NA months), see Fig. 3B. There was also no significant difference based on localization (p = 0.3, median OS for siNET 87.4, for panNET 171.6, and for other 113.4 months) and based on grading (p = 0.3, median OS for NET G1 106.8, for NET G2 71.1, and for NET G1/G2 unclassified 113.4 months). The median OS from diagnosis was 137.1 months (95% CI 113.4–181.4 months), with 44 deaths observed in the follow-up period.

### Adverse events

With regards to adverse events (AEs), no AEs were documented in 89/129 patients (69.0%), while 29 (22.5%) had one AE and 11 patients (8.5%) had at least two AEs. The 50 instances of AEs of these 40 patients (31.0%) are summarized in Table 3. The most common side effects were diarrhea and abdominal pain, which together accounted for 38%. In one person, lanreotide was reduced to 90 mg due to sweating, but at the same time the application interval was shortened to 21 days. In another case, SSA was discontinued due to a possibly drug-related psychosis (on octreotide) and given stable disease of the NET G1. Of the six patients with SSA re-initiation, all but one patient had no AEs documented (diarrhea in one case).

**Table 3:** Adverse events

Adverse events (AEs)	Frequency (n = 129)	Percentage of patients	Percentage of all instances of AEs (n = 50)
No AEs	89	69.0	
Any AEs	40	31.0	
Diarrhea	11	8.5	22
Abdominal pain	8	6.2	16
Nausea, vomiting	5	3.9	10
Exocrine pancreatic insufficiency	5	3.9	10
Hyperglycemia	4	3.1	8
Weight loss	4	3.1	8
Sweating	3	2.3	6
Flatulence	3	2.3	6
Headache	2	1.6	4
Fatigue	2	1.6	4
Recurrent hypertension	1	0.8	2
Vertigo	1	0.8	2
Psychosis	1	0.8	2

## Discussion

The objective of this retrospective analysis was to compare the PFS and OS of patients with well-differentiated gastroenteropancreatic NET who received either octreotide LAR or lanreotide autogel. This study included 129 patients treated at the Medical University of Vienna between 2010 and 2020, with a median follow-up of 92.0 months (7.7 years) for octreotide and 47.3 months (4 years) for lanreotide. Our long-term experience with SSA demonstrated no statistically significant difference in PFS or OS between the two treatment cohorts. There were differing preferences concerning the areas of application for the two SSA, which corresponded to the different study conditions and their approval status.

In line with the respective pivotal trials, current guidelines recommend octreotide and lanreotide as a first-line antiproliferative therapy for advanced/metastatic enteropancreatic NET with SSTR expression and a Ki-67 of up to 10% / slow growth [5, 25]. PROMID was a phase IIIB study that included 85 treatment-naïve patients with NET from the midgut (small intestine and right colon) [26], of whom most had low hepatic tumor burden (64/85 with ≤ 10%) and G1 disease (81/85 with Ki-67 ≤ 2%) and some (33/85) had carcinoid syndrome [12]. Conversely, the phase III CLARINET trial enrolled 204 enteropancreatic NET patients with no hormonal syndromes and a Ki-67 < 10%, with many (33%) showing a liver tumor volume > 25% [13]. Furthermore, almost all patients (96%) in CLARINET had stable disease at baseline [13]. Given these obvious differences, direct comparisons of the two study populations appear challenging.

### Progression-free survival

In our cohort, the median PFS with octreotide was 36.0 months (95% CI 23.2–68.2 months) and the median PFS with lanreotide 29.8 months (95% CI 18.7–48.5 months), with the numerical difference not meeting statistical significance (p = 0.8). At 24 months of follow-up, 61.5% of patients receiving octreotide and 54.7% of patients given lanreotide were progression-free. No significant differences emerged when stratified by tumor grade (NET G1 versus NET G2) or primary tumor localization (small intestine versus pancreas). For instance, the median PFS for lanreotide in panNET was 20.6 months (versus 25.2 months with octreotide), and the median PFS for octreotide in siNET was 37.7 months (versus 37.6 months with lanreotide). Notwithstanding the observational study design and different application preferences with varying subgroup sizes, octreotide and lanreotide seemed equal in their disease-stabilizing activity.

In contrast to our results, the median time to tumor progression in PROMID was only 14.3 months with octreotide versus 6 months with placebo (hazard ratio [HR] 0.34, 95% CI 0.20–0.59) [12]. On the other hand, median PFS was not reached with lanreotide in the CLARINET core study (versus 18.0 months with placebo, HR 0.47, 95% CI 0.30–0.73) but subsequently reported as 38.5 months in the CLARINET open-label extension (OLE) study [13, 27]. The reasons for these striking discrepancies in PFS between the two trials have been previously discussed and include different response assessment criteria (WHO versus RECIST) and a different tumor biology of the recruited patients [13, 14]. Importantly, compared to our study, CLARINET did not allow rapidly progressing NET to be enrolled (the Ki-67 index had to be < 10% and most tumors were stable), whereas this was not required in our study (10% of patients had a Ki-67 index > 10%) [13]. Further differences relate to tumor grade (52% NET G2 in our small intestinal NET cohort versus < 5% in PROMID) and functional status (54% hormonally active in our cohort versus 0% in CLARINET and 70% in the small intestinal NET subgroup of our study versus 39% in PROMID) [12, 13]. Of further note, the treatment protocols (SSA every 28 days) and imaging intervals (e.g., every 12 weeks in CLARINET initially) were clearly defined in these two clinical trials, while there was more variation in our retrospective real-world analysis depending on the individual clinical case.

Beyond this, comparative evidence regarding the antitumor efficacy of lanreotide and octreotide is scant. A Spanish registry study called GETNE-TRASGU (n = 535) reported a median PFS of 30.1 months (95% CI 23.1–38.0 months) for lanreotide and of 28.0 months (95% CI 22.1–32.0 months) for octreotide, with a HR of 0.90 (95% CI 0.71–1.12) indicating similar effectiveness [16]. A retrospective study from 2022 involving 105 patients also found no significant difference (p = 0.2665) between octreotide (median PFS of 12 months, 95% CI 6–18 months) and lanreotide (median PFS of 10.8 months, 95% CI 6–15.6 months) [15]. In a US drug claims database study (n = 548), lanreotide and octreotide had a similar (p = 0.6094) time to index SSA discontinuation (median of 198 days and 228 days, respectively) [28]. In contrast, a comparable US study of administrative claims data (n = 762) found longer treatment durations for lanreotide patients (median of 41.3 versus 26.8 months, p = 0.004) [29].

In our study, NET G2 emerged as a clear adverse prognostic factor for PFS (HR of 1.98 in univariate and 1.78 in multivariable Cox regression analysis). Correspondingly, the treatment benefit of SSA is less clear in NET patients with a high proliferative activity (Ki-67 index > 10%) [11, 30], and the median PFS of those patients was only 12.8 months versus 34.1 months in patients with a lower Ki-67 index. Univariate Cox regression also indicated that history of surgery might be associated with a more favorable prognosis, even though a causal relationship cannot be established on the basis of these data. Patients receiving SSA as a first line had a longer PFS than patients on later-line SSA (p = 0.063, median PFS of 36.0 versus 18.4 months). Hence, the fact that 22/129 patients (17%) were not on first-line SSA should also be considered when interpreting this analysis. Moreover, restarting SSA after disease progression upon SSA or another therapy resulted in a median PFS of 8.7 months in six patients, which may suggest an additional benefit. Of note, CLARINET FORTE found a median PFS of 8.3 months (siNET) and of 5.6 months (panNET) in patients receiving lanreotide 120 mg every 14 days after progressing on standard-dose lanreotide [31].

### Overall survival

In our analysis, there was no statistically significant difference (p > 0.9) between octreotide and lanreotide with regards to overall survival, although the median OS figures alone (90.3 months and 113.4 months, respectively) might initially suggest differences. Based on these findings, the choice between octreotide and lanreotide does not appear to impact overall survival of NET patients. However, due caution is necessary since several potentially confounding variables were not addressed. Likewise, in the PROMID trial, there was no OS difference between patients randomized to octreotide and those taking placebo (84.7 versus 83.7 months) – yet, this was only the secondary endpoint, and cross-over may have influenced the results [14]. In GETNE-TRASGU, median OS was 85.9 months for octreotide and 85.0 months for lanreotide [16]. For reference, data from the Surveillance, Epidemiology, and End Results (SEER) program showed a median OS of 16.2 years for NET G1 and 8.3 years for NET G2 (in our cohort, median OS from diagnosis was 11.4 years) [2].

### Adverse events

Retrospective collection of adverse events showed an excellent tolerability of SSA in our cohort, which is in line with previous findings [12, 13]. No AE were documented in 69.0% of our patients, and common gastrointestinal AE such as diarrhea, abdominal pain, and nausea/vomiting occurred in < 10% of patients. Moreover, most patients (> 82.2%) received the standard antiproliferative doses of SSA that were established by the two pivotal trials as 120 mg (lanreotide) or 30 mg (octreotide) every 4 weeks. The recommended doses were targeted in patients who started on a lower dose.

Overall, there do not appear to be any major differences between octreotide and lanreotide in terms of adverse events and feasibility [11]. Concerning safety and individual choice of treatment, there are differences in the administration of these drugs (deep subcutaneous injection for lanreotide and intramuscular injection for octreotide) [11], which may lead to different patient or physician preferences. Importantly, intramuscular administration is discouraged in patients at high risk for bleeding. There is also a study suggesting that missed intramuscular injections (radiologically evident as subcutaneous nodules) are common with octreotide, with these nodules observed in 44% of patients receiving octreotide and occurring more frequently in obese patients (not associated with a worse outcome in this study) [32]. Apart from that, the two pivotal trials PROMID and CLARINET performed health-related quality-of-life assessments and found comparable scores between the two study arms [12, 13].

### Limitations

When interpreting these results, several limitations must be recognized. Retrospective analyses such as this are prone to selection or information bias and can be subject to confounding. As this is an observational study, radiological tumor assessment was performed at different intervals and was based on routine imaging reports that were, however, regularly reviewed in tumor boards in equivocal cases. The single-center approach might render these data less generalizable than results from multicenter or international studies, but the detailed and uniform data collection represents a strength. This study was exploratory in nature, and neither sample size calculations nor multiple testing corrections were performed – larger studies may in theory detect differences in survival between subgroups.

### Conclusion

Given the findings of our analysis and the results of previous studies, octreotide and lanreotide seem to be equally effective for the treatment of NET, so that a switch to the other preparation may be appropriate in the event of supply shortages or application problems with one preparation. As of now, however, the respective pivotal studies support different areas of application (octreotide for intestinal NET and lanreotide for enteropancreatic NET in the advanced or metastatic setting), hence lanreotide and octreotide should be preferentially used according to their approved indication. A prospective trial comparing the efficacy of both SSA is required for definite conclusions. Further studies could also investigate potential differences between the two drugs in terms of combinations therapy, maintenance treatment, high-dose use, or application in highly proliferative NET. Ultimately, this could refine therapeutic strategies for NET patients.

## Data Availability

Enquiries for further data can be directed to the corresponding author.
